# DBBP: database of binding pairs in protein-nucleic acid interactions

**DOI:** 10.1186/1471-2105-15-S15-S5

**Published:** 2014-12-03

**Authors:** Byungkyu Park, Hyungchan Kim, Kyungsook Han

**Affiliations:** 1 Institute for Information and Electronics Research, Inha University, Incheon, South Korea; 2 Department of Chemistry, Inha University, Incheon, South Korea; 3 School of Computer Science and Engineering, Inha University, Incheon, South Korea

## Abstract

**Background:**

Interaction of proteins with other molecules plays an important role in many biological activities. As many structures of protein-DNA complexes and protein-RNA complexes have been determined in the past years, several databases have been constructed to provide structure data of the complexes. However, the information on the binding sites between proteins and nucleic acids is not readily available from the structure data since the data consists mostly of the three-dimensional coordinates of the atoms in the complexes.

**Results:**

We analyzed the huge amount of structure data for the hydrogen bonding interactions between proteins and nucleic acids and developed a database called DBBP (**D**ata**B**ase of **B**inding **P**airs in protein-nucleic acid interactions, http://bclab.inha.ac.kr/dbbp). DBBP contains 44,955 hydrogen bonds (H-bonds) of protein-DNA interactions and 77,947 H-bonds of protein-RNA interactions.

**Conclusions:**

Analysis of the huge amount of structure data of protein-nucleic acid complexes is labor-intensive, yet provides useful information for studying protein-nucleic acid interactions. DBBP provides the detailed information of hydrogen-bonding interactions between proteins and nucleic acids at various levels from the atomic level to the residue level. The binding information can be used as a valuable resource for developing a computational method aiming at predicting new binding sites in proteins or nucleic acids.

## Background

Protein-nucleic acid interactions play an important role in many biological activities. Site-specific DNA-binding proteins or transcription factors (TFs) play important roles in gene regulations by forming protein complexes [1]. These protein-DNA complexes may bind alone or in combination near the genes whose expression they control [2]. For example, DNA-binding proteins may regulate the expression of a target gene [1], so protein-DNA interactions are important for DNA replication, transcription and gene regulations in general.

Protein-RNA interactions also have important roles in a wide variety of gene expression [3]. For instance, ribonucleoprotein particles (RNPs) bind to RNA in the post-transcriptional regulation of gene expression [4], and tRNAs bind to aminoacyl-tRNA synthetases to properly translate the genetic code into amino acids [5]. As protein and RNA mutually interact, RNA-binding proteins are essential molecules in degradation, localization, regulating RNA splicing, RNA metabolism, stability, translation, and transport [6]. Therefore, identification of amino acids involved in DNA/RNA binding or (ribo)nucleotides involved in amino acid binding is important for understanding of the mechanism of gene regulations.

As the number of structures of protein-DNA/RNA complexes that have been resolved has been increased plentifully for the past few years, a huge amount of structure data is available at several databases [7-10]. However, the data on the binding sites between proteins and nucleic acids is not readily available from the structure data, which consist mostly of the three-dimensional coordinates of the atoms in the complexes. A recent database called the Protein-RNA Interface Database (PRIDB) [9] provides the information on protein-RNA interfaces by showing interacting amino acids and ribonucleotides in the primary sequences. However, it does not provide the binding sites on the interacting partners of the amino acids and ribonucleotides in protein-RNA interfaces.

In this study we performed wide analysis of the structures of protein-DNA/RNA complexes and built a database called DBBP (**D**ata**B**ase of **B**inding **P**airs in protein-nucleic acid interactions). The database shows hydrogen-bonding interactions between proteins and nucleic acids at an atomic level, which is not readily available in any other databases, including the Protein Data Bank (PDB) [11]. The binding pairs of hydrogen bonds provided by the database will help researchers determine DNA (or RNA) binding sites in proteins and protein binding sites in DNA or RNA molecules. It can also be used as a valuable resource for developing a computational method aiming at predicting new binding sites in proteins or nucleic acids. The rest of the paper presents the structure and interface of the database.

## Materials and methods

### Protein-DNA/RNA complexes

The protein-DNA/RNA complexes determined by X-ray crystallography were selected from PDB. As of February, 2013 there were 2,568 protein-DNA complexes and 1,355 protein-RNA complexes in PDB. After extracting complexes with a resolution of 3.0 Å or better, 2,138 protein-DNA complexes (called the DS1 data set) and 651 protein-RNA complexes (the DS2 data set) remained.

### Binding sites in protein-nucleic acid interactions

Different studies [9,12-14] have defined slightly different criteria for a binding site in protein-nucleic acid interactions. For example, in RNABindR [15,16] and BindN [17] an amino acid with an atom within a distance of 5 Å from any other atom of a ribonucleotide was considered to be an RNA-binding amino acid.

As for the criteria for a binding site between proteins and nucleic acids, we use a hydrogen bond (H-bond), which is stricter than the distance criteria. The locations of hydrogen atoms (H) were inferred from the surrounding atoms since hydrogen atoms are invisible in purely X-ray-derived structures. H-bonds between proteins and nucleic acids were identified by finding all proximal atom pairs between H-bond donors (D) and acceptors (A) that satisfy the following the geometric criteria: (1) the hydrogen-acceptor (H-A) distance *<*2.5 Å, (2) the donor-hydrogen-acceptor (D-H-A) angle *>*90°, (3) the contacts with the donor-acceptor (D-A) distance *<*3.9 Å, (4) H-A-AA angle *>*90°, where AA is an acceptor antecedent. These are the most commonly used criteria for H bonds. In particular, the criteria of H-A distance *<*2.5 Å and D-H-A angle *>*90° are essential for H bonds [18]. If there is no H-bond within a protein-nucleic acid complex, we eliminated the complex from the data sets of DS1 and DS2. As a result, we gathered 2,068 protein-DNA complexes (DS3) and 637 protein-RNA complexes (DS4).

**Figure 1:**
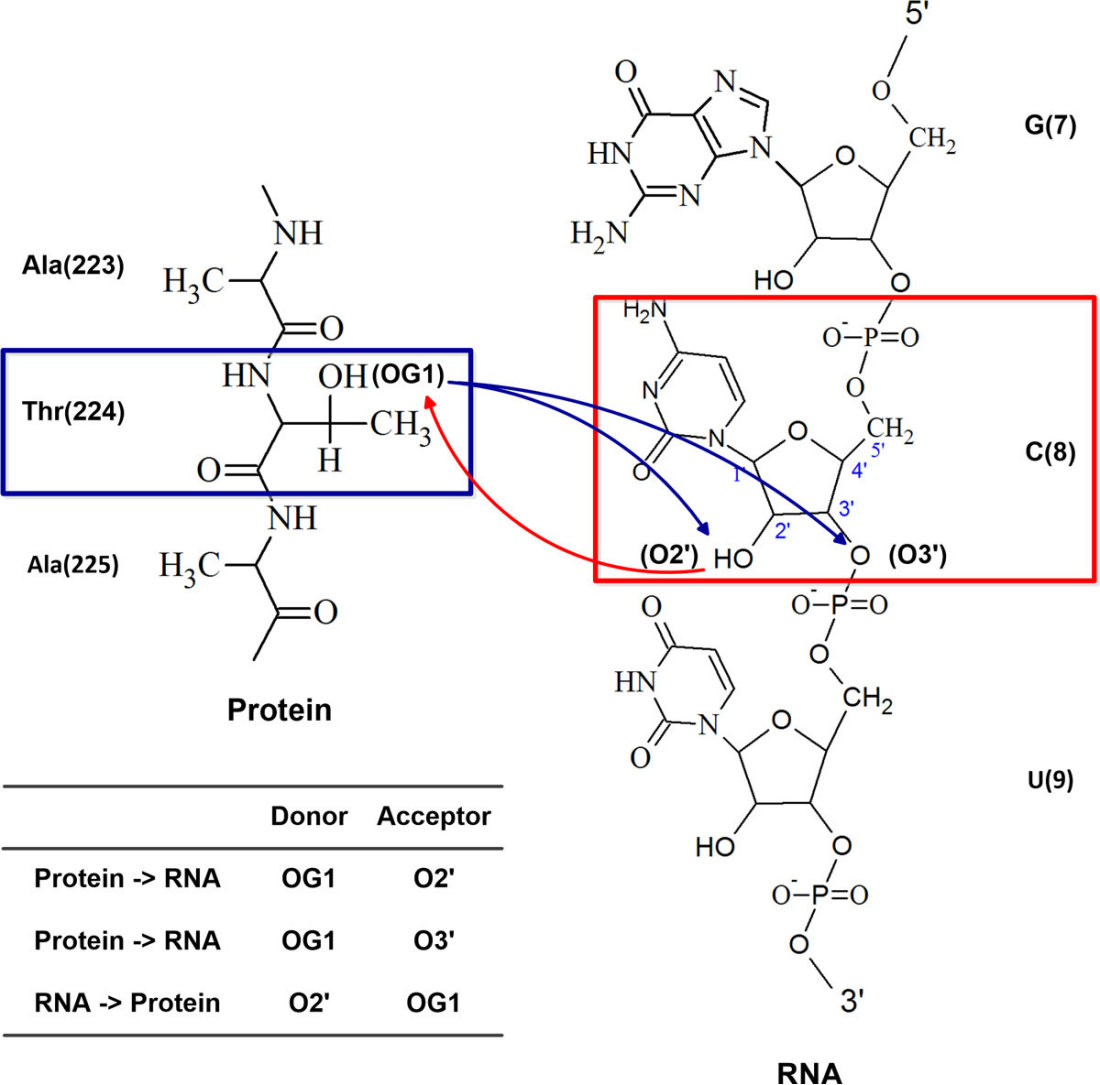
**Three H-bonds between Cytosine (C8) and Threonine (Thr224)**. Three H-bonds between Cytosine (C8) and Threonine (Thr224) of a protein-RNA complex (PDB ID: 4F3T). O2′ of Cytosine donates hydrogen to OG1 of Threonine. OG1 of Threonine donates hydrogen to O2′ of Cytosine and OG1 of Threonine donates hydrogen to O3′ of Cytosine.

**Figure 2:**
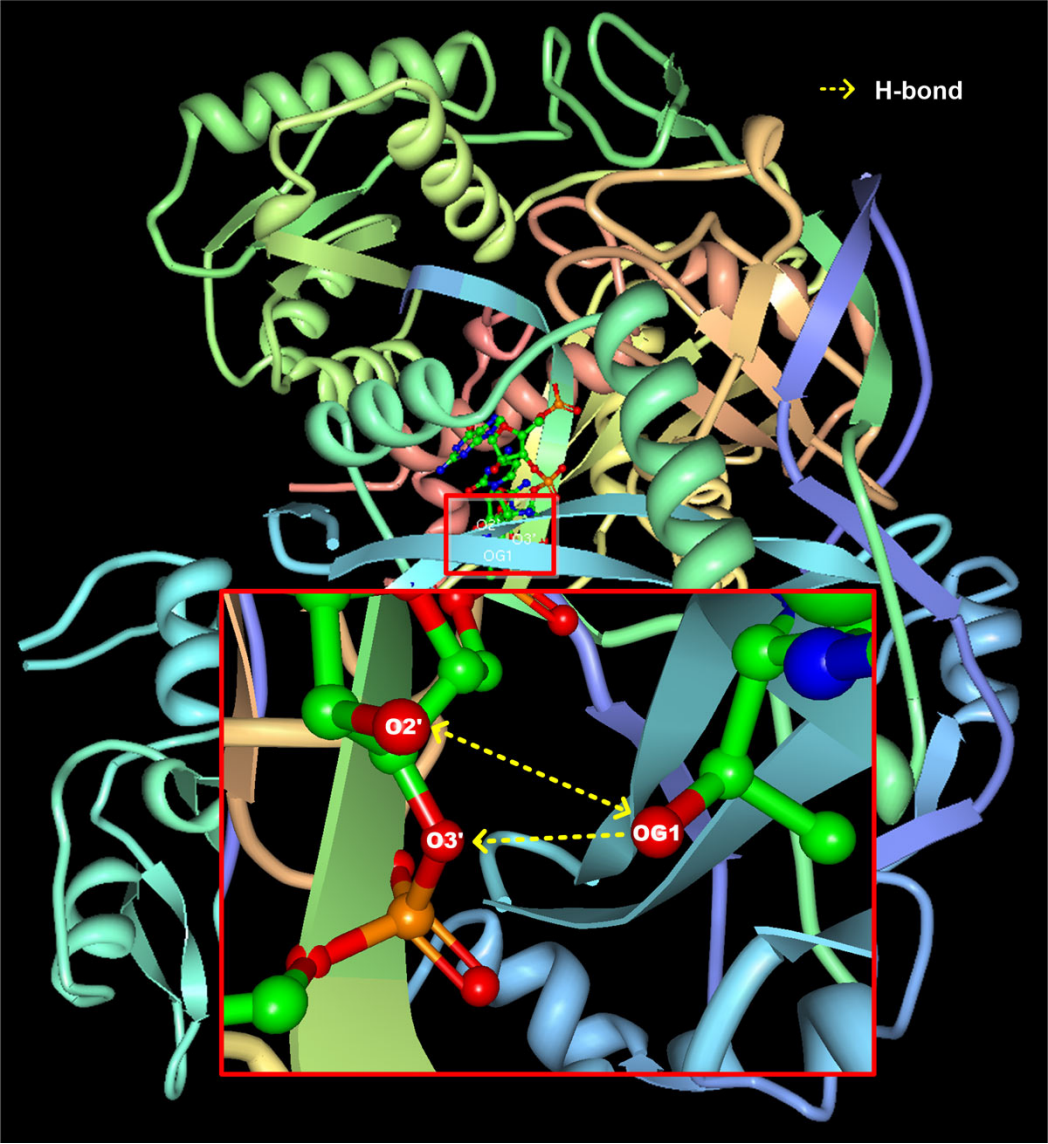
**The structure of a protein-RNA complex (PDB ID: 4F3T)**. The enlarged box shows three hydrogen bonds between Cytosine and Threonine. O2′ donates hydrogen to OG1. OG1 donates hydrogen to O2′ and O3′.

As an example, Figure 1 shows three H-bonds between Threonine (Thr224) and Cytosine (C8) in a protein-RNA complex (PDB ID: 4F3T) [19]. In protein-RNA interactions, OG1 and N of Threonine can act as a hydrogen donor and OG1 and O of Threonine can act as a hydrogen acceptor. N3, N4, O2′ and O3′ of Cytosine can act as a hydrogen donor and N3, O2, O2′, O3′, O4′, O5′, OP1 and OP2 of Cytosine can act as a hydrogen acceptor. In this example, Cytosine is the 8th nucleotide in RNA chain R and Threonine is the 224th amino acid in protein chain A. OG1 of Threonine donates hydrogen to O2′ of Cytosine, OG1 of Threonine donates hydrogen to O3′ of Cytosine, and O2' of Cytosine donates hydrogen to OG1 of Threonine. Figure 2 shows the structure of the protein-RNA complex (PDB ID: 4F3T).

### The probability of binding amino acid

Let *P *(+) be the probability that an amino acid is a binding site and *P (−*) be the probability that an amino acid is a non-binding site in protein-nucleic acid interactions (Equations 1 and 2).

(1) $$P\left(+\right)=\frac{\text{DNA/RNA - binding amino acids}}{\text{amino acids in protein - DNA/RNA complexes}}$$

(2) $$P\left(-\right)=\frac{\text{DNA/RNA - non - binding amino acids}}{\text{amino acids in protein - DNA/RNA complexes}}$$

Then, the conditional probability *P(A*|+) is the probability that the binding amino acid is A. Likewise, the conditional probability *P(A|−*) is the probability that the non-binding amino acid is A. Equation 5 is the log-likelihood ratio of *P(A*|+) and *P(A|−*).

(3) $$P\left(A|+\right)=\frac{P\left(A\cap +\right)}{P\left(+\right)}$$

(4) $$P\left(A|-\right)=\frac{P\left(A\cap +\right)}{P\left(-\right)}$$

(5) $$log-likelihoodratio=log_{2}\frac{P\left(A|+\right)}{P\left(A|-\right)}$$

## Results and discussion

### Hydrogen bonds in protein-nucleic acid interactions

We obtained H-bonds from 2,068 protein-DNA complexes (DS3) and 637 protein-RNA complexes (DS4) using HBPLUS [18,20] with the H-bond criteria: $\bar{HA}<2.5\overset{\circ }{A}$, ∠*DHA >*90°, $\bar{DA}<3.9\overset{\circ }{A}$. There are a total of 44,955 H-bonds in protein-DNA complexes and 77,947 H-bonds in protein-RNA complexes. Table 1 shows the number of atoms, which are occurrences in H-bonds of amino acids. In the 44,955 H-bonds of protein-DNA complexes, there are 41,298 hydrogen donors and 3,657 hydrogen acceptors in amino acids. In the 77,947 H-bonds of protein-RNA complexes, there are 59,796 hydrogen donors and 18,151 hydrogen acceptors in amino acids. Table 2 shows the number of atoms, which are occurrences in H-bonds of (ribo)nucleotides. In the 44,955 H-bonds of protein-DNA complexes, there are 3,657 hydrogen donors and 41,298 hydrogen acceptors in DNAs. In the 77,947 H-bonds of protein-RNA complexes, there are 18,151 hydrogen donors and 59,796 hydrogen acceptors in RNAs.

**Table 1:** Atoms of amino acids involved in H-bonding interactions with nucleic acids.

		RNA-protein complex			DNA-protein complex		
AA	Atom	Acceptor	Donor	#H-bonds	Acceptor	Donor	#H-bonds
Ala	N		1,069	1,653		674	808
	O	567			134		
	OXT	17					

Arg	NH2		9,252	22,395		6,144	13,705
	NH1		7,278			4,665	
	NE		4,011			2,191	
	N		1,388			606	
	O	455			99		
	OXT	13					

Asn	ND2		3,268	4,953		2349	3,119
	OD1	934			408		
	N		549			261	
	O	202			101		

Asp	OD2	1,416		2,829	353		735
	OD1	1,183			290		
	O	178			31		
	N		52			61	

Cys	SG	23	76	125	19	120	215
	O	24					
	N		2			76	

Gln	NE2	168	2496	4,468	2	1,593	2,571
	OE1	1,108			363	521	
	N		480				
	O	216			92		

Glu	OE2	1,691		3,507	275		737
	OE1	1,315			260		
	O	193			19		
	N		308			183	

Gly	N		1,518	2,699		1749	1,902
	O	1,175			153		
	OXT	6					

His	NE2	412	1,454	3,591	30	768	1,254
	ND1	536	1,014		15	327	
	N		106			90	
	O	69			24		

Ile	N		258	309		433	466
	O	40			33		
	OXT	11					

Leu	N		507	766		362	387
	O	259			25		

Lys	NZ		9,864			5,145	6,351
	N		852	11,436	86	1,120	
	O	717					
	OXT	3					

Met	SD	105		662	15		147
	O	276			13	119	
	N		278				
	OXT	3					

Phe	O	333		539	42		247
	N		206			205	

Pro	O	161		161	28		28

Ser	OG	1,179	4,675	6,997	182	3,533	4,741
	N		683			958	
	O	460			68		

Thr	OG1	1,058	4,406	7,267	158	3,017	4,252
	O	750			132		
	N		1,053			945	

Trp	NE1		532	582		358	393
	OXT	16					
	O	14			10		
	N		20			25	

Tyr	OH	597	1,935	2,682	133	1,800	2,511
	O	93			28		
	N		57			550	

Val	O	174		326	36		386
	N		151			350	
	OXT	1					
		18,151	59,796	77,947	3,657	41,298	44,955

**Table 2:** Atoms of nucleotides involved in H-bonding interactions with amino acids.

		RNA-protein complex			DNA-protein complex		
Nucleotide	Atom	Acceptor	Donor	#H-bonds	Acceptor	Donor	#H-bonds
A	N1	402	140	22,103	58	23	10,254
	N3	1,071	79		748	26	
	N6		1,472			621	
	N7	505			580		
	O2'	4,240	4,269				
	O3'	1,711	86		361	100	
	O4'	252			276		
	O5'	110			188		
	OP1	1,754			4,039		
	OP2	6,012			3,234		

C	N3	335	49	16,189	127	3	9,502
	N4		785			1,272	
	O2	2,556			959		
	O2'	2,101	2,209		1	1	
	O3'	1,150	56		257	139	
	O4'	663			209		
	O5'	117			118		
	OP1	5,176			3,858		
	OP2	992			2,558		

G	N1	547	759	30,350	2	204	14,864
	N2		3,907			761	
	N3	655	53		399	2	
	N7	1,660			2,238		
	O2'	2,047			2,383		
	O3'	1,031	24		438	157	
	O4'	450			420		
	O5'	585			197		
	O6	2,396			2,272		
	OP1	10,523			4,359		
	OP2	3,330			3,415		

U/T	N3	173	386	9,305	29	234	10,335
	O2	1,561			1,165		
	O2'	1,310	1,445				
	O3'	1,067	49		351	114	
	O4	1,199			796		
	O4'	166			257		
	O5'	45			216		
	OP1	1,108			3,548		
	OP2	796			3,625		

59,796			18,151	77,947	41,298	3,657	44,955

If an atom of DNA acts as a hydrogen acceptor, an atom of protein should be a hydrogen donor. Hence, the number of DNA acceptors (41,298) is the same as the number of protein donors (41,298), and the number of DNA donors (3,657) is the same as the number of protein acceptors (3,657). Likewise, the number of RNA acceptors (59,796) is the same as the number of protein donors (59,796) and the number of RNA donors (18,151) is the same as the number of protein acceptors (18,151).

**Figure 3:**
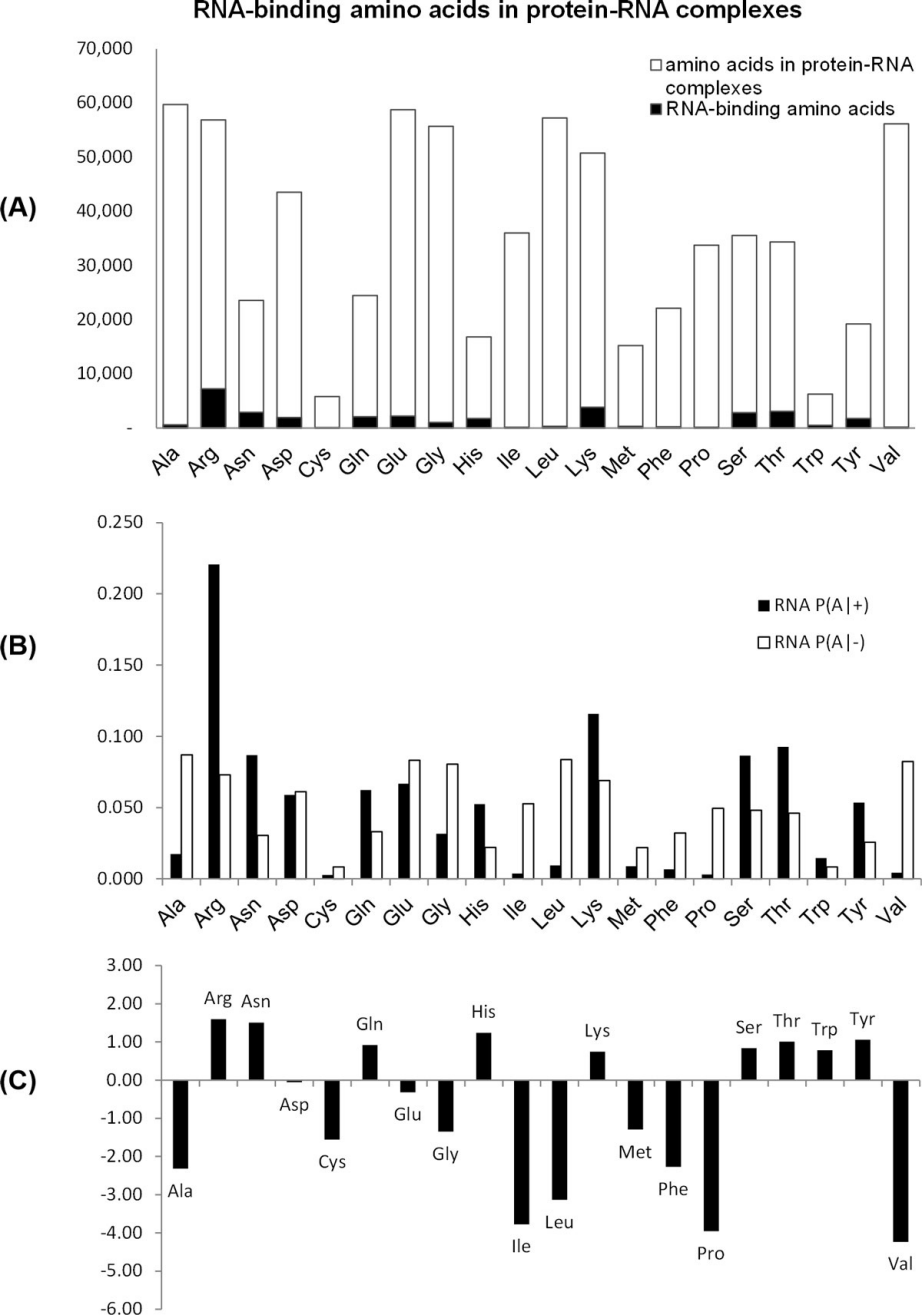
**RNA-binding amino acids in protein-RNA complexes**. (A) Amino acids in the protein-RNA complexes and RNA-binding amino acids. (B) The probability that the binding amino acid is A (*P(A*|+)) and the probability that non-binding amino acid is A (*P(A|−*)). (C) The log-likelihood ratio *log*_2_(*P(A*|+)*/P(A|−*)).

Figure 3 shows RNA-binding amino acids in protein-RNA complexes. Ala, Arg, Glu, Gly, Leu, Lys, and Val are more frequent than others in protein-RNA complexes (Figure 3A). In binding sites with RNA, Arg has the most frequently observed amino acid. Figure 3C shows the log-likelihood ratio (Equation 5) for each amino acid. Amino acids with a positive log-likelihood ratio have a higher chance to bind to RNA than those with a negative log-likelihood ratio. Arg has the highest log-likelihood ratio (1.59), and Val has the lowest log-likelihood ratio (-4.24). Interestingly, Ala has a negative log-likelihood ratio although it is frequently observed in protein-RNA complexes. This is because Ala is rarely observed in binding sites.

**Figure 4:**
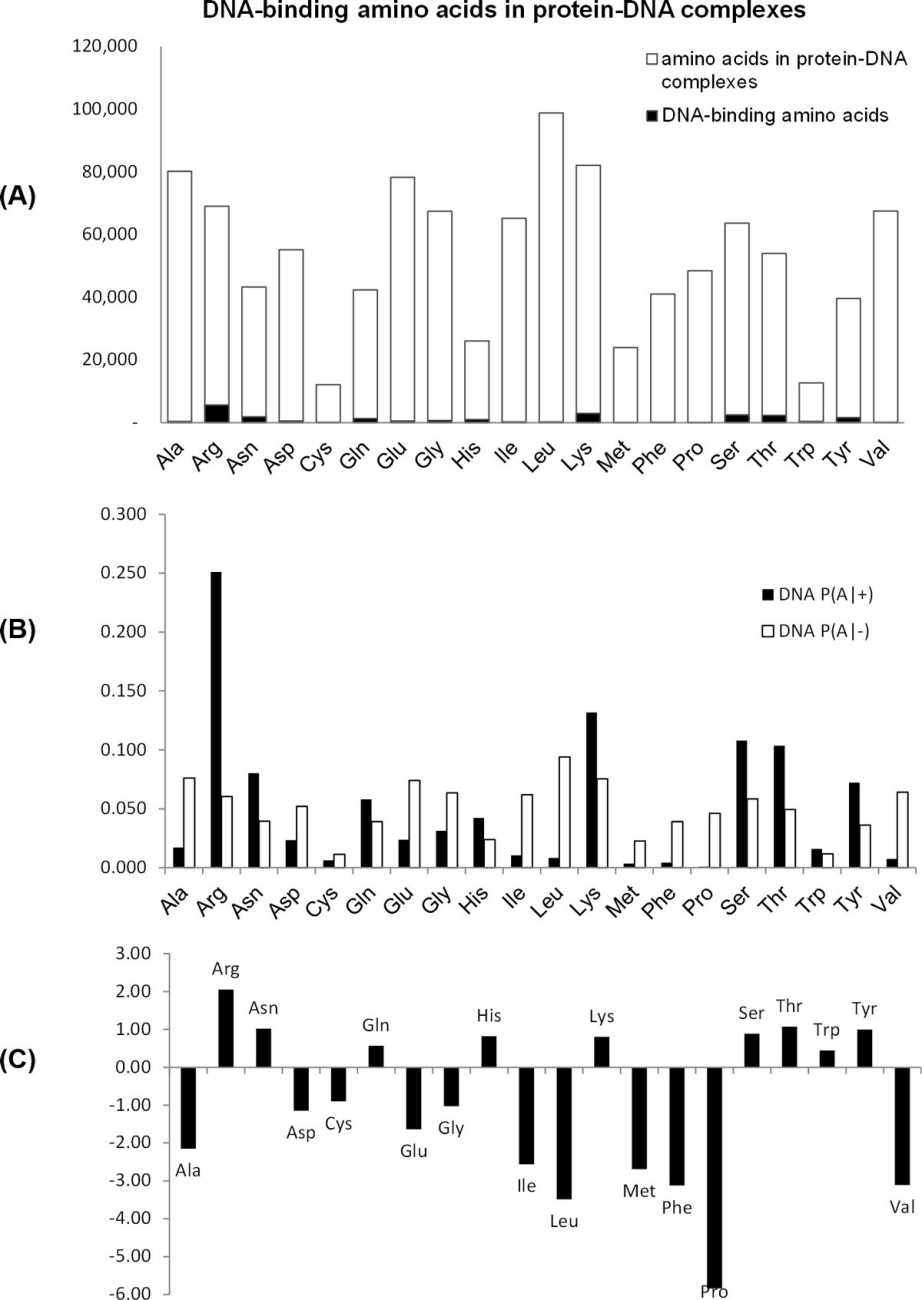
**DNA-binding amino acids in protein-DNA complexes**. (A) Amino acids in the protein-DNA complexes and DNA-binding amino acids. (B) The probability that the binding amino acid is A (*P(A*|+)) and the probability that non-binding amino acid is A (*P(A|−*)). (C) The log-likelihood ratio *log*_2_(*P(A*|+)*/P(A|−*)).

Figure 4 shows DNA-binding amino acids in protein-DNA complexes. Ala, Arg, Glu, Gly, Leu, Lys, Ser, and Val are more frequent than others in protein-DNA complexes (Figure 4A). As in protein-RNA interactions, Arg has the most frequently observed amino acid in the binding sites with DNA.

### Web interface

DBBP shows binding pairs at various levels, from the atomic level to the residue level. When it shows detailed information on H-Bonds, it shows the donors and acceptors of each H-bond. A same type of atom can play a role of hydrogen donor or acceptor depending on the context. We generated XML files for binding sites of protein-DNA/RNA complexes. Users of the database can access the XML file via PDB ID.

**Figure 5:**
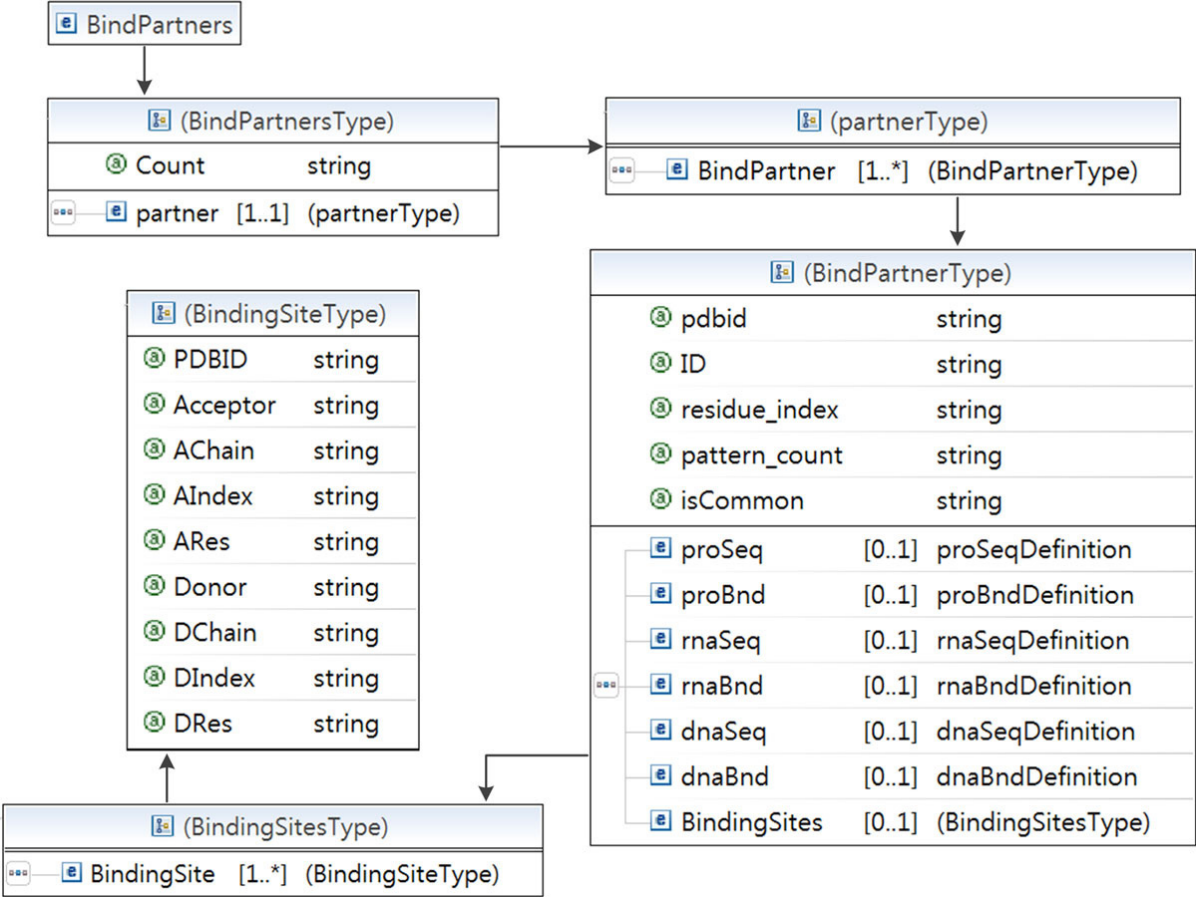
**The XML schema of the database**. XML files were generated for the binding sites in protein-DNA complexes and protein-RNA complexes via the XML schema.

Figure 5 shows our XML schema. The BindPartner element has elements and attributes, which are PDB ID, protein sequence (proSeq), protein bond (proBnd), DNA/RNA sequence (dnaSeq, rnaSeq), and DNA/RNA bond (dnaBnd, rnaBnd). DNA/RNA and protein bonds represent binding site '+' and non-binding site '-'. The BindingSite element has attributes, which are PDBID, Acceptor, Acceptor chain, Acceptor index, Acceptor residue, Donor, Donor chain, Donor index, and Donor residue.

## Conclusion

From an extensive analysis of the structure data of protein-DNA/RNA complexes extracted from PDB, we have identified hydrogen bonds (H-bonds). Analysis of the large amount of structure data for H-bonds is labor-intensive, yet provides useful information for studying protein-nucleic acid interactions. The protein-DNA complexes contain 44,955 H-bonds, which have 3,657 hydrogen acceptors (HA) and 41,298 hydrogen donors (HD) in amino acids, and 41,298 HA and 3,657 HD in nucleotides. The protein-RNA complexes contain 77,947 H-bonds, which have 18,151 HA and 59,796 HD in amino acids, and 59,796 HA and 18,151 HD in nucleotides. Using the data of H-bonding interactions, we developed a database called DBBP (**D**ata**B**ase of **B**inding **P**airs in protein-nucleic acid interactions). DBBP provides the detailed information of H-bonding interactions between proteins and nucleic acids at various levels. Such information is not readily available in any other databases, including PDB, but will help researchers determine DNA (or RNA) binding sites in proteins and protein binding sites in DNA or RNA molecules. It can also be used as a valuable resource for developing a computational method aiming at predicting new binding sites in proteins or nucleic acids. The database is available at http://bclab.inha.ac.kr/dbbp.

## Authors' contributions

Byungkyu Park implemented the databse and prepared the first draft of the manuscript. Hyungchan Kim drew figures and prepared the manuscript together. Kyungsook Han supervised the work and rewrote the manuscript. All authors read and approved the final manuscript.
